# Autophagy-mediated NKG2D internalization impairs NK cell function and exacerbates radiation pneumonitis

**DOI:** 10.3389/fimmu.2023.1250920

**Published:** 2023-11-24

**Authors:** Ruiqing Wang, Xinyue Ma, Xinyu Zhang, Dizhi Jiang, Hongyuan Mao, Zerun Li, Yu Tian, Bo Cheng

**Affiliations:** Qilu Hospital of Shandong University, Cheeloo College of Medicine, Shandong University, Jinan, China

**Keywords:** radiation pneumonitis, CXCL10/CXCR3, autophagy, NKG2D, NK cell

## Abstract

**Introduction:**

Radiation pneumonitis is a critical complication that constrains the use of radiation therapy for thoracic malignancies, leading to substantial morbidity via respiratory distress and lung function impairment. The role of Natural killer (NK) cells in inflammatory diseases is well-documented; however, their involvement in radiation pneumonitis is not fully understood.

**Methods:**

To explore the involvement of NK cells in radiation pneumonitis, we analyzed tissue samples for NK cell presence and function. The study utilized immunofluorescence staining, western blotting, and immunoprecipitation to investigate CXCL10 and ROS levels, autophagy activity, and NKG2D receptor dynamics in NK cells derived from patients and animal models subjected to radiation.

**Result:**

In this study, we observed an augmented infiltration of NK cells in tissues affected by radiation pneumonitis, although their function was markedly diminished. In animal models, enhancing NK cell activity appeared to decelerate the disease progression. Concomitant with the disease course, there was a notable upsurge in CXCL10 and ROS levels. CXCL10 was found to facilitate NK cell migration through CXCR3 receptor activation. Furthermore, evidence of excessive autophagy in patient NK cells was linked to ROS accumulation, as indicated by immunofluorescence and Western blot analyses. The association between the NKG2D receptor and its adaptor proteins (AP2 subunits AP2A1 and AP2M1), LC3, and lysosomes was intensified after radiation exposure, as demonstrated by immunoprecipitation. This interaction led to NKG2D receptor endocytosis and subsequent lysosomal degradation.

**Conclusion:**

Our findings delineate a mechanism by which radiation-induced lung injury may suppress NK cell function through an autophagy-dependent pathway. The dysregulation observed suggests potential therapeutic targets; hence, modulating autophagy and enhancing NK cell activity could represent novel strategies for mitigating radiation pneumonitis.

## Introduction

Radiotherapy (RT) is pivotal in the treatment of malignant tumors. Nonetheless, radiation pneumonitis (RP) stands out as a frequent clinical side effect observed in thoracic radiotherapy patients. This condition significantly impacts the long-term survival rates and prognoses of cancer patients, constituting a primary impediment to the efficacy of radiotherapy (1, 2). Consequently, the suppression of RP-associated inflammatory infiltration and incidence reduction represent pressing clinical imperatives. Given the intricate nature of its progression mechanism, enhanced prognostic outcomes may hinge on innovative therapeutic approaches stemming from an improved comprehension of RP’s progression mechanism.

The progression of radiation pneumonitis is influenced by various factors, including alterations in the tumor and inflammatory microenvironments (3, 4). Natural killer (NK) cells constitute a vital component of the inflammatory microenvironments and play a pivotal role in inflammatory responses. On one hand, they possess the capability to directly eliminate pathogen-infected cells, thereby preventing further dissemination. On the other hand, they can secrete an array of cytokines and chemical factors, such as tumor necrosis factor-alpha (TNF-α), interferon-gamma (IFN-γ), and interleukins (such as IL-10 and IL-13), to modulate the inflammatory response (5, 6). The equilibrium between signals from activating and inhibitory receptors governs the functional outcomes of NK cells. Unlike B and T cell antigen receptors, NK cell receptors are encoded in the germline and do not undergo somatic recombination, enabling them to mount rapid responses in inflammatory reactions (7, 8). NKG2D, serving as a primary activating receptor on the surface of NK cells, primarily recognizes and binds to specific ligands expressed by stressed, infected, or cancerous cells, which are typically absent on healthy cell surfaces. Upon binding to these ligands, NKG2D activates the NK cell, resulting in the destruction of the target cell (9–11). However, the role of NK cells in the progression of RP has not been investigated to date.

NK cell function is primarily regulated by chemotactic factors in inflammatory environments (12). During influenza virus infection, previous research by Wareing MD et al. found that CXCR2 plays a critical role in recruiting neutrophils to the lungs, leading to their accumulation in lung tissue. Interestingly, this accumulation does not significantly contribute to virus clearance, offering valuable insights into the varying impacts of chemokines on the quantity and functionality of immune cells (13). Recent studies suggest that inflammation can trigger the release of numerous chemokines, including IL-8, which attract neutrophils and result in the production of additional reactive oxygen and nitrogen species, consequently impairing neutrophil function (14). Our study revealed that CXCL10/CXCR3 activation in radiation pneumonitis leads to increased NK cell infiltration, while the accumulation of reactive oxygen species (ROS) causes excessive autophagy, thereby inhibiting NK cell function. Delving deeper into these specific mechanisms is crucial for gaining a better understanding of NK cells’ role in lung injury.

Autophagy is an intracellular biological process responsible for degrading and recycling cellular components, frequently triggered by ROS (15). This self-regulating mechanism aids in preserving normal cellular functions when moderately engaged. However, if excessively stimulated, it can cause irreparable harm to the cells (16). The process of autophagy comprises several sequential steps. Initially, an autophagosome engulfs materials earmarked for degradation, such as damaged proteins or organelles. Subsequently, the autophagosome fuses with a lysosome inside the cell, leading to the degradation and recycling of the contents encapsulated within the autophagosome. Autophagy plays a pivotal role in various biological processes, such as defending against infections, delaying the aging process, responding to hunger, and managing stress reactions (17–19).

In this study, we observed an elevated count of NK cells concurrent with a decrease in their functionality during radiation pneumonia. Mechanistically, radiation-induced lung injury results in the release of CXCL10, which activates CXCR3 on the surface of NK cells, resulting in the intracellular accumulation of ROS. Elevated ROS levels constitute the primary cause of excessive autophagy in NK cells during radiation pneumonia. Subsequently, autophagy initiates the internalization of NKG2D and its degradation via the lysosomal pathway, leading to a deterioration in NK cell function and exacerbating the advancement of radiation pneumonia. Our findings, which confirm the involvement of NK cells in radiation pneumonia, may provide valuable insights for the clinical diagnosis and treatment of this condition.

## Methods

### Cell culture

NK92 cells (ATCC^®^, CRL-2407™) were maintained in MEM-α (#12571063, GIBCO, USA) supplemented with 10% FBS, 10% horse serum, 1% non-essential amino acid, 1% pen-strep, 1% sodium pyruvate, 0.1 mM 2-β-mercaptoethanol, 0.2 mM myo-inositol, and 2.5 μM folic acid. Cells were maintained at 37 °C at 5% CO2 levels. For conditioning, cells were cultured for approximately 2 months and were routinely checked for mycoplasma infection.

The mouse lung cancer cell line CMT167 was obtained from the European Collection of Authenticated Cell Cultures (ECACC) and cultured in Dulbecco’s modified Eagle’s medium (DMEM; Life Technologies, USA), containing 10% (v/v) fetal bovine serum (FBS; Life Technologies, USA).

### Animal

Animal studies were approved by Institutional Animal Care and Use Committee of Qilu Hospital affiliated to Shandong University. The mice were bred in our air-conditioned animal facility and housed with a 12/12 hr light/dark cycle and with ad libitum access to food and water. In the survival study, the animals were observed daily. Animals displaying symptoms, such as severe hunchback posture, apathy, decreased motion, or activity, dragging legs, unkempt fur, or drastic loss of body weight were killed by cervical dislocation. Excised tumor tissues were further examined through hemtoxylin and eosin, and immunofluorescence staining.

Luciferase-expressing CMT167 cell lines (5 x 10^5^ cells in 40 μL PBS) were injected through the chest wall into the lung of C57BL/6 (5-week-old; SPF Biotechnology Co., Ltd, Beijing, China) female mice on day 0. Depleting antibodies were NK1.1 clone PK136 (#BE0036, BioXCell), and IgG2a isotype control (#BE0085, BioXCell). Tumor growth was examined at 5, 10, and 15 days after inoculation via bioluminescence imaging (IVIS spectrum *in vivo* imaging system, PerkinElmer, USA). On day 5, mice were randomly assigned to each treatment group.

### Western blotting

The RIPA (#89901, Thermo Fisher Scientific, USA) buffer were used to lyse the harvested NK92 cells on ice about 30 min and centrifuged at 4 °C and 17,000g for 50 min. The BCA protein assay kit (#P0010, Beyotime Institute of Biotechnology, China) was used for the protein concentration measurement. Subjected the samples to SDS-PAGE electrophoretically, and then transferred to PVDF membranes. All membranes were blocked by Tween-Tris-buffered saline containing 5% non-fat milk for 2 h at room temperature and then incubated with primary antibodies as follows: GAPDH (#ab9485, Abcam), β-tublin (#ab179511, Abcam), AP2A1 (#ab189995, Abcam), AP2M1(#ab75995, Abcam), LAMP2 (#ab13524, Abcam), LC3 (#ab62721, Abcam), NKG2D (#ab36136, Abcam), CXCR3 (#ab288437, Abcam). At room temperature, all the membranes were washed by TBST three times, and then incubated with horseradish peroxidase II antibody (#ZB-2301, #ZB-2305, ZSGB-BIO, China) for 1 hour. The synergistic chemical imager (ECL) kit (#34096, Thermo Fisher Scientific, USA) was used for staining protein gels and then used the manufacturer’s ChemImager 5500 V2.03 software scan.

### Co-IP

Co-Immunoprecipitations were used to detected the interaction between proteins. Briefly, the RIPA (#89901, Thermo Fisher Scientific, USA) buffer were used to lyse the harvested cell on ice about 60 min and corresponding antibodies were incubated with the Protein A/G Magnetic beads for immunoprecipitation (#B23201, Bimake, USA) on ice for 30 min. Cell lysates were centrifuged, and the supernatants were added to and incubated with the protein A/G Magnetic beads (above incubated with corresponding antibodies) under rotation at 4°C overnight. After being washed three times with high-salt buffer. The beads were boiled for 7 min with 2 × SDS sample buffer, followed by Western blotting with corresponding antibodies.

### PCR

Total RNA was extracted from cells using TRIzol reagent (#10296010CN, Invitrogen, USA) and reverse-transcribed using the Rever Tra Ace qPCR RT Kit (#FSQ-101, Toyobo, Japan). cDNA was amplified using SYBR Green on the Roche Light Cycler 480 for quantification. The relative expression levels of mRNA were normalized to glycer- aldehyde-3-phosphate dehydrogenase (GAPDH). Sequences of the primers used are shown in **Supplementary Table 1**.

### HE staining

HE staining was performed using a HE Staining Kit (#G1120, Solarbio, China). Briefly, staining of brain sections was carried out using Mayers hematoxylin, followed by eosin. Following eosin staining for 50 s, and dehydration by ethanol (95, 100%), the sections were cleared by xylene and mounted. These images were obtained using Nikon’s confocal microscope (Nikon, Japan). We then calculated the proportion of radiation pneumonium area in the total area of the lobes.

### Multiplex immunofluorescence staining

For multiplex immunofluorescence staining, we followed the Opal protocol staining method for the following markers: NK1.1 (#ab234107, Abcam), LC3B (#ab192890, Abcam), LysoTracker (Invitrogen. #L12492), and NKG2D (Abcam, #ab302907). All sections were cover-slipped using Anti-Fade Fluorescence Mounting Medium (#ab104135, Abcam). Phenochart software was used to perform a 20X (0.5 μm/pixel) scan analysis of selected tissue areas after scanning by Vectra Polaris Automated Quantitative Pathology Imaging System.

Staining images were evaluated by two blinded pathologists, with the intensity of autophagy scored from 0 to 3, with 0 (no LC3 staining), 1 (weakly LC3 staining), 2 (moderately LC3 staining), and 3 (severely LC3 staining). The positive rate of LC3 in NK cells was also scored using a scale of 0-3: 0 (0-9%), 1 (10%-25%), 2 (26%-50%), 3 (51%-75%), and 4 (76%-100%). The autophagy intensity score and the positive cell rate score are then multiplied, and the product is used as the final autophagy score.

### 3D-SIM

In our study, we use 3D structured illumination microscopy (3D-SIM), a super-resolution imaging technique, to enhance our understanding of NK cells. To begin with, specimens were prepared following standard fixation and staining procedures suitable for 3D-SIM. Once prepared, the specimens were placed under the 3D-SIM microscope, equipped with a high-numerical-aperture lens. Subsequently, these raw images were processed through a reconstruction algorithm. Live, three-color, 3D-SIM imaging was performed on the OMX-Flex system (GE Healthcare).

### Flow cytometry

To detect apoptosis, cells were rinsed with PBS, incubated with Annexin V-FITC and PI (#556547, BD Biosciences, USA) at room temperature for 15 min. Apoptosis results were analyzed on a C6 flow cytometer (BD Biosciences, USA). Data were analyzed in Accuri 6C software analysis.

Intracellular ROS production was detected using MitoSOX™ Red (#M36009, MitoSox, Invitrogen). And cell staining of single-cell suspensions was performed using the following fluorophore-conjugated antibodies: CD45 (#157214, Biolegend, USA), NK1.1 (#156506, Biolegend, USA), IFN-γ (#505808, Biolegend, USA), TNF-α (#506306, Biolegend, USA), Granzyme B (#372208, Biolegend, USA), NKG2D (#115711, Biolegend, USA).

### Statistical analysis

The unpaired two-tailed Student’s t-test was used to compare differences between two groups. Multiple comparisons were performed with one-way ANOVA using Dunnett’s multiple comparisons test. Survival curves were estimated by the Kaplan–Meier method and compared using the log-rank test. Statistical analysis was conducted using GraphPad Prism (Version 8.0). P < 0.050 was considered statistically significant for all the two-sided tests.

## Results

### Radiation pneumonia is accompanied by an accumulation of NK cells and a decline in their functionality

To investigate changes in NK cell quantity and function during radiation pneumonitis, we induced radiation pneumonitis in 6-8-week-old C57/BL6 mice by exposing them to a single 20 Gy dose of radiation. Following irradiation, we collected lung tissue samples and observed an increase in both lung volume and weight, consistent with the potential development of pulmonary edema observed in clinical patients after radiation exposure. This observation aligns with the potential development of pulmonary edema observed in clinical patients after radiation exposure. The increased lung volume and weight signify fluid accumulation in lung tissue, a characteristic feature of pulmonary edema. These results imply that the mice exhibited a physiological response to radiation-induced lung injury similar to that observed in humans (**Supplementary Figure 1A**).

**Figure 1:**
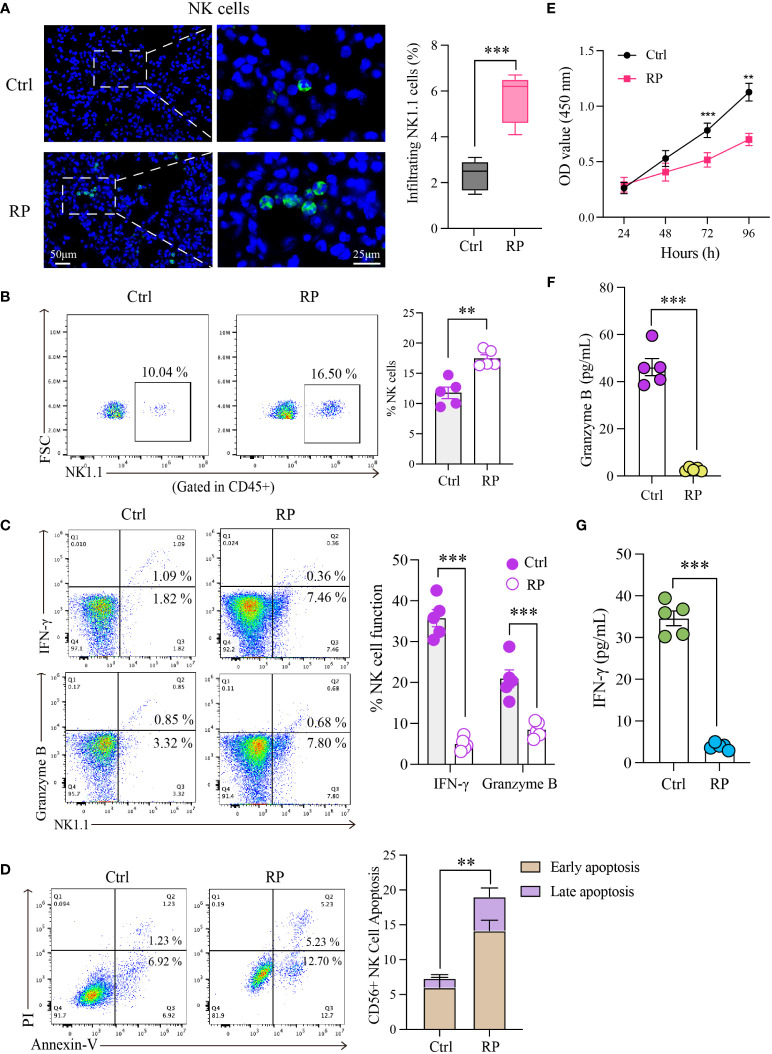
Radiation pneumonia is accompanied by an accumulation of NK cells and a decline in their functionality. Immunofluorescence staining **(A)** and flow cytometry analysis **(B)** for detecting the number of NK cells. Scale bar (left) = 50 μm, Scale bar (right) = 25 μm. **(C)** Flow cytometry analysis for the expression of functional indicators (IFN-γ and granzyme B) of lung tissue-infiltrating NK cells. **(D, E)** The apoptosis rate and CCK8 results indicated that the vitality of NK cells was significantly weaker when mice had a radiation pneumonia. **(F, G)** The levels of IFN-γ and granzyme B in the culture supernatant of NK cells. **(A–C, F, G)**: n = 5; **(D, E)**: n = 3. Each point represents an individual experiment. *, P < 0.050; **, P < 0.010; ***, P < 0.001. ***P = 0.0003 **(A)**, **P = 0.001 **(B)**, ***P < 0.0001 IFN-γ, ***P = 0.0008 Gran B **(C)**, **P = 0.0023 **(D)**, ***P = 0.0002 72h, **P = 0.0016 96h **(E)**, ***P < 0.001 **(F)**, ***P < 0.001 **(G)**.

Hematoxylin and eosin (HE) staining revealed significant inflammatory infiltration in the lungs on the 14th day after irradiation, confirming the successful model establishment. Building on this foundation, we euthanized the mice on the 14th day and isolated pulmonary mononuclear cells for quantifying and assessing NK cell function. The results revealed a significant increase in the proportion of NK cells in the lungs of the RP group compared to the control group (non-irradiated mice) (**Figures 1A, B**). Subsequently, we assessed the expression of IFN-γ and granzyme B in NK cells using flow cytometry. Surprisingly, despite the increase in NK cell numbers, the function of NK cells in the RP group’s lungs was significantly diminished compared to the control group (**Figure 1C**).

To gain a clearer understanding of NK cell status, we isolated and cultured NK cells obtained from the lungs of radiation pneumonitis mice using magnetic bead separation. The extracellular apoptosis rate of NK cells in the lungs of the RP group was higher than that in the control group (**Figure 1D**). Additionally, CCK8 results demonstrated significantly reduced vitality of NK cells in mice with radiation pneumonia (**Figure 1E**). Subsequently, we cultured the isolated NK cells *in vitro* and assessed the levels of IFN-γ and granzyme B in the culture supernatant after 48 hours. The results revealed a substantial reduction in the RP group’s lung NK cells’ ability to secrete cytokines, indicating a significant functional difference compared to the control group (**Figures 1F, G**). These findings indicate inconsistent changes in the number and function of NK cells in radiation pneumonitis, marked by an increase in infiltration but functional exhaustion.

### Lung injury promotes the recruitment of NK cells via the CXCL10/CXCR3 pathway

**Figure 2:**
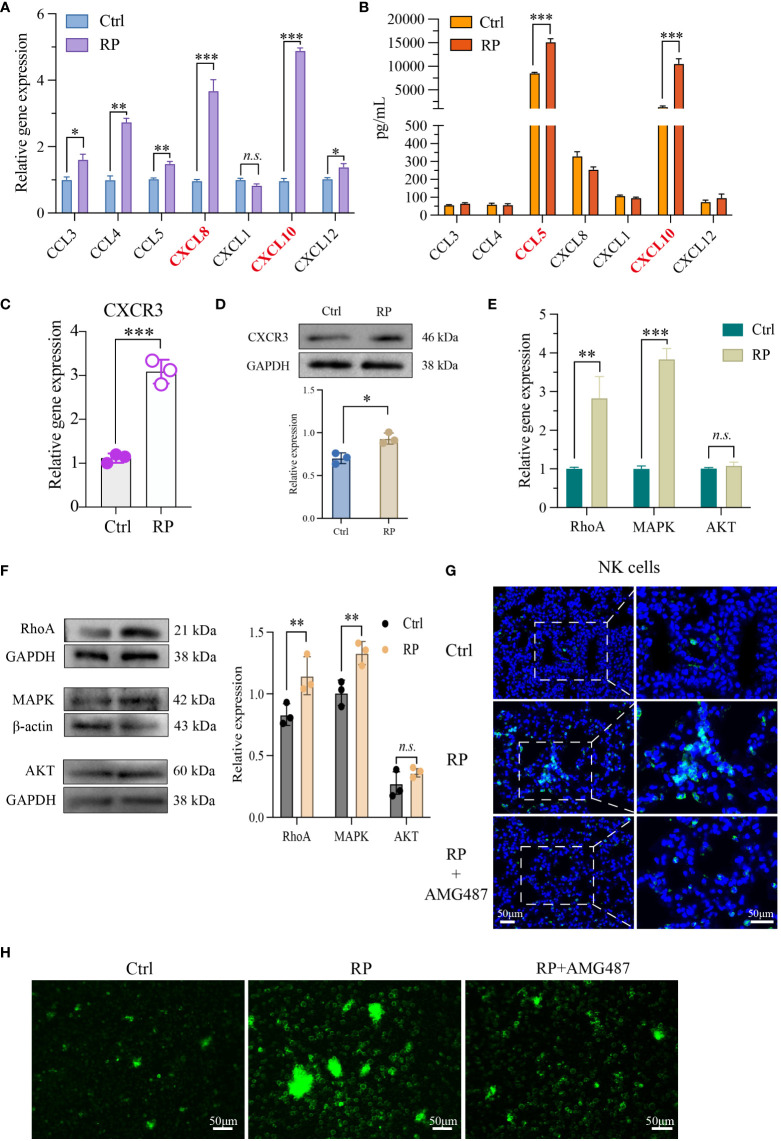
Lung injury promotes the recruitment of NK cells via the CXCL10/CXCR3 pathway. NK cell-associated chemokines in mouse lung tissue **(A)** and BALF **(B)** were detected respectively. PCR **(C, E)** as well as Western blotting analysis **(D, F)** revealed that NK cells from the lungs of RP group were markedly activated at both the RNA and protein levels. **(G)** Demonstration of AMG487 reversing radiotherapy-induced decline in NK cell count in mice. Scale bar (left) = 50 μm, Scale bar (right) = 50 μm. **(H)** Transwell assay depicting NK cell migration under control, radiotherapy, and radiotherapy with AMG487. Scale bar = 50 μm **(A-G)**: n = 3. Each point represents an individual experiment. *, P < 0.050; **, P < 0.010; ***, P < 0.001. *P = 0.0142 CCL3, **P = 0.0033 CCL4, **P = 0.0090 CCL5, ***P = 0.0002 CXCL8, ***P = 0.0005 CXCL10, *P = 0.0358 CXCL12 **(A)**, ***P < 0.0001 CCL5, ***P = 0.0001 CXCL10 **(B)**, ***P = 0.0003 **(C)**, *P = 0.0118 **(D)**, **P = 0.0048 RhoA, ***P< 0.0001 MAPK **(E)**, **P = 0.0044 RhoA, **P = 0.0038 MAPK **(F)**.

In our previous research, we identified substantial alterations in both the quantity and function of NK cells in radiation pneumonitis (RP). To explore the underlying mechanisms, PCR and ELISA were used to quantify the levels of NK cell-associated chemokines in mouse lung tissue and bronchoalveolar lavage fluid (BALF), respectively. In lung tissue, we noted a significant elevation in CXCL8 and CXCL10 levels in the RP group (**Figure 2A**). Conversely, in the BALF, CCL5 and CXCL10 exhibited a substantial increase in the RP group (**Figure 2B**). Additionally, we collected peripheral blood samples from patients undergoing the same radiation regimen and divided them into two groups based on the presence or absence of radiation pneumonitis development. ELISA results reveal a significant increase in CXCL10 levels in the peripheral blood supernatant of patients who developed radiation pneumonitis (**Supplementary Figure 1B**). Given that CXCL10 displayed significant changes in both groups, we hypothesized that CXCL10 may play an important role in radiation pneumonitis.

Next, we assessed the activation status of CXCR3, the receptor for CXCL10, in NK cells. PCR and Western blot findings indicated significant activation of CXCR3 at both the RNA and protein levels in NK cells from the lungs of RP group mice (**Figures 2C, D**). To further investigate the chemotactic capacity<city/> of NK cells induced by the activation of CXCL10/CXCR3 in radiation pneumonitis, we examined the expression of cell migration-related molecules MAPK, RhoA, and AKT. The results show that in radiation pneumonitis, the transcription and protein levels of MAPK and RhoA in NK cells have increased, but the changes in AKT are not significant (**Figures 2E, F**). Immunofluorescence and transwell assays indicate that the CXCR3 inhibitor AMG487 significantly reduces the migratory capacity<city/> of NK cells after radiation exposure, leading to a decrease in NK cell infiltration (**Figures 2G, H** and **Supplementary Figures 1F, G**). In summary, we hypothesize that the release of CXCL10 induced by radiation activates the CXCR3 receptors on the surfaces of NK cells, thereby promoting their infiltration into lung tissues.

### Overactive autophagy in lung NK Cells induced by radiation

**Figure 3:**
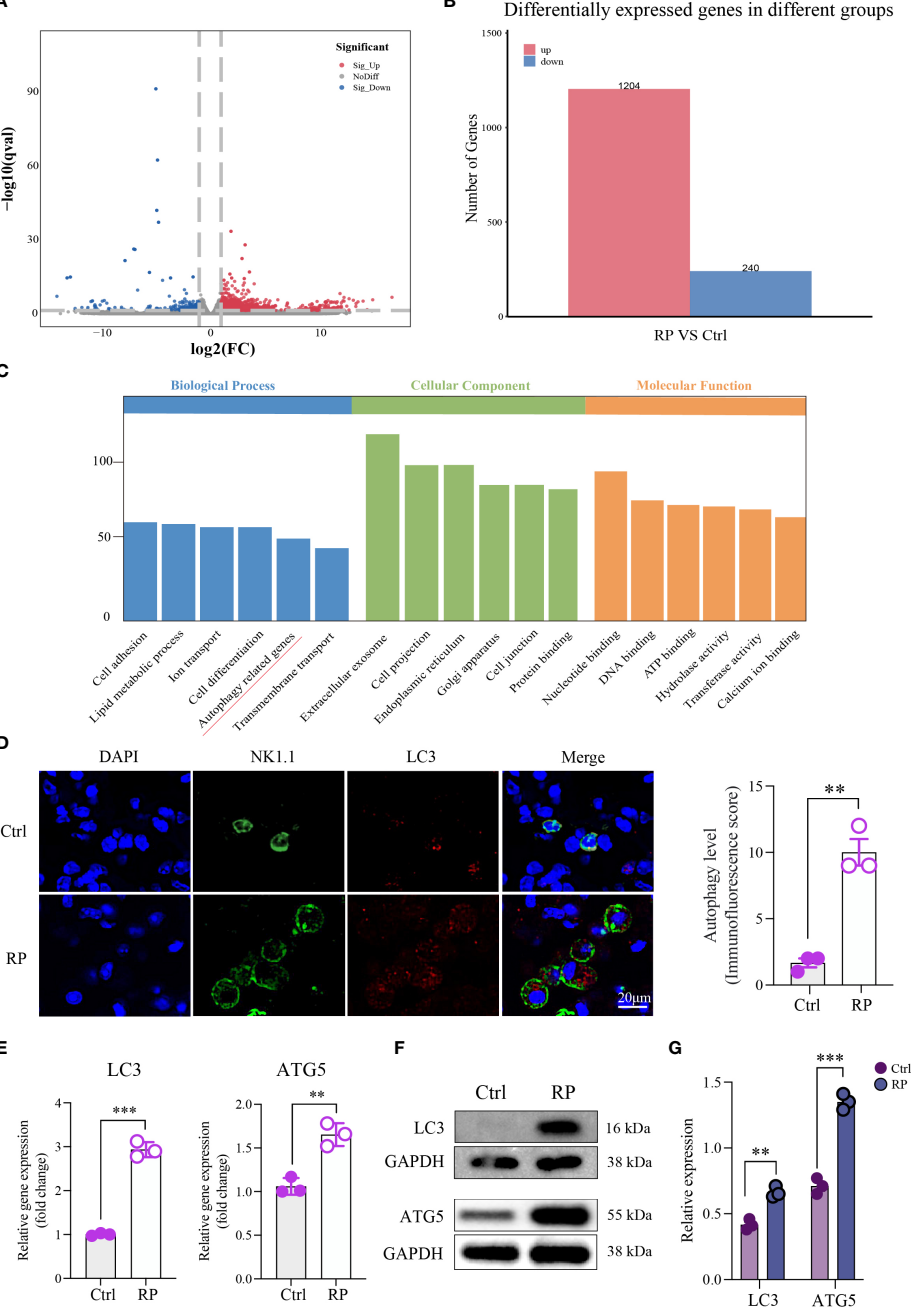
Overactive Autophagy in Lung NK Cells Induced by Radiation. **(A)** Volcano Plot of Differentially Expressed Genes: This plot shows the relationship between gene expression levels and statistical significance, highlighting genes that are differentially expressed between two conditions. The x-axis represents the log-transformed expression levels of genes, the y-axis represents the negative logarithm of statistical significance, and the scatter plot represents differentially expressed genes. Red dots indicate upregulated genes, blue dots indicate downregulated genes, and gray dots indicate genes with no significant difference. **(B)** Number of Differentially Expressed Genes: Comparison of the number of differentially expressed genes detected in RNA-seq analysis under different conditions. The numbers in the table represent the count of genes significantly upregulated or downregulated in each condition. **(C)** Pathway enrichment analysis of the differentially expressed gene set using the KEGG database. This plot shows the differentially expressed genes enriched KEGG pathways. **(D)** Compared to the control group, multiplex immunofluorescence staining showed that autophagy was excessively activated in the lung NK cells of the RP group mice. Scale bar = 20 μm. **(E–G)** RT-qPCR and Western blotting results indicated an increase in the expression of autophagy-related proteins in lung NK cells from the RP group mice. **(D–G)**: n = 3. Each point represents an individual experiment. *, P < 0.050; **, P < 0.010; ***, P < 0.001. **P = 0.0014 **(D)**, ***P < 0.001 LC3, **P = 0.0031 ATG5 **(E)**, **P = 0.0018 LC3, ***P = 0.0002 ATG5 **(G)**.

To better understand the mechanism of NK cell function suppression in radiation pneumonitis, we conducted RNA-seq analysis comparing NK cells from mice with radiation pneumonitis to those from healthy lungs. The results revealed significant transcriptomic differences between NK cells from the two groups of mice (**Figures 3A, B**). Subsequent enrichment analysis using the KEGG pathway indicated that the differentially expressed genes were predominantly associated with the autophagy pathway (**Figure 3C**).

Immunofluorescence staining showed that the level of autophagy in lung NK cells was increased in the RP group compared with the control group (**Figure 3D**). RT-qPCR and Western blot results indicated elevated expression of autophagy-related proteins in lung NK cells from mice in the RP group (**Figures 3E–G**). These findings confirm that autophagy occurs in NK cells within the radiation pneumonia microenvironment, and further investigation is required to elucidate the downstream mechanism.

### Restoration of NK cell function through inhibition of ROS accumulation or excessive autophagy

**Figure 4:**
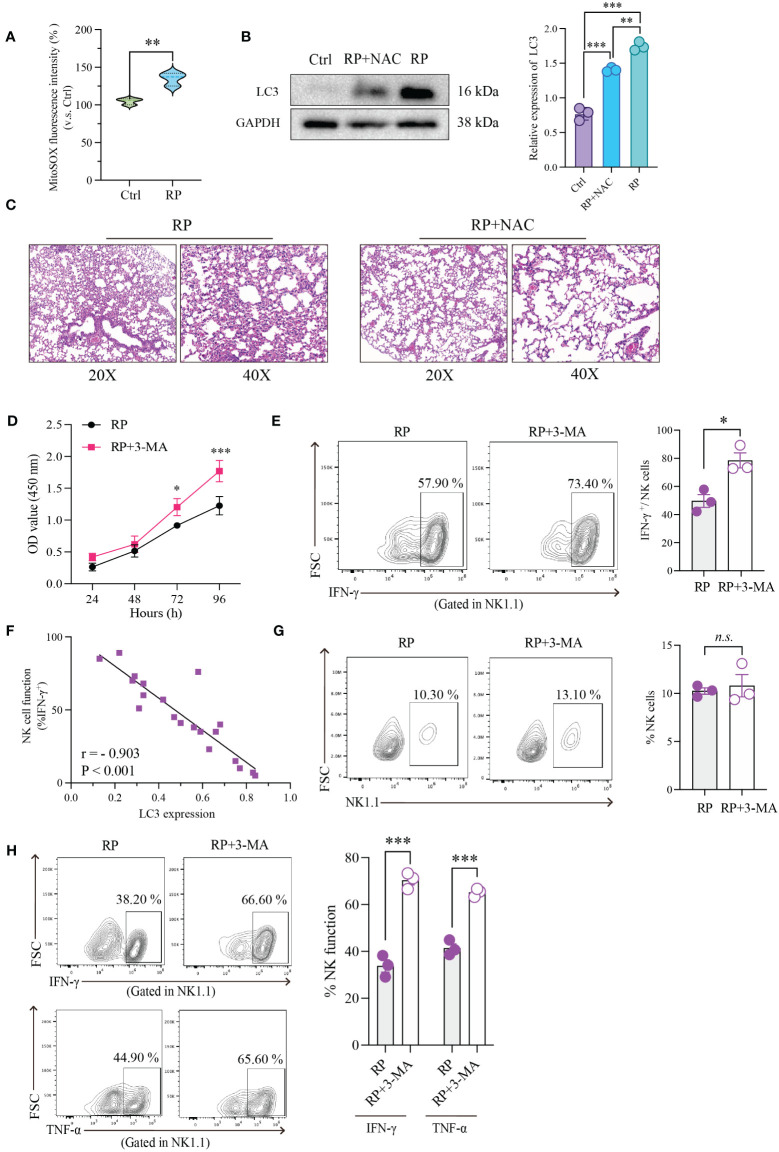
Restoration of NK Cell Function through Inhibition of ROS Accumulation or Excessive Autophagy. **(A)** Using mitochondrial ROS-specific fluorescent dye (MitoSOX red) to observe the distribution of mitochondrial ROS. **(B)** The effect of NAC on the expression of autophagy-related protein (LC3) in NK cells as demonstrated by Western blotting. **(C)** HE-staining of lung tissue from mice exposed to 20Gy irradiation with or without autophagy inhibitor treatment. Left: 20x magnification. Right: 40x magnification. ROS inhibition reduces radiation-induced lung damage. **(D)** As demonstrated by CCK8 assays, radiation-induced excessive autophagy of NK cells was inhibited by 3-MA, resulting in significantly higher proliferation rates than those in control group. **(E)** Flow cytometry showed that NK cell function was significantly restored after its autophagy was inhibited. **(F)** Linear correlation analysis confirmed that there was a significant negative correlation between NK cell function and LC3 expression level. **(G)** Flow cytometry showed that under the influence of 3-MA, the number of lung-infiltrating NK cells didn’t show significant changes. **(H)** The RP+3-MA group significantly outperformed the RP-only group in terms of NK cell function. **(A–H)**: n = 3. Each point represents an individual experiment. *, P < 0.050; **, P < 0.010; ***, P < 0.001. **P = 0.0058 **(A)**, ***P < 0.0001 Ctrl vs RP+NAC, ***P < 0.0001 Ctrl vs RP, **P = 0.0019 RP vs RP+NAC **(B)**, *P = 0.0253 72h, ***P < 0.0001 96h **(D)**, *P = 0.0151 **(E)**, ***P = 0.0004 IFN-γ, ***P = 0.0003 TNF-α **(H)**.

Numerous studies have shown that the buildup of reactive oxygen species (ROS) during inflammation is a major driver of excessive cellular autophagy. Consequently, we quantified ROS levels in lung tissues from mice with radiation pneumonitis and healthy mice. The results revealed a substantial increase in ROS accumulation in the radiation pneumonitis group (**Figure 4A**). ROS can accumulate through various mechanisms, and based on prior reports on other inflammatory conditions, CXCL10/CXCR3 activation can also contribute to ROS buildup. Accordingly, we conducted relevant experiments. However, in radiation pneumonitis, the primary source of ROS is radiation-induced cellular damage. These data are presented for reference purposes.

Our findings revealed an upward trend in mitochondrial ROS production in the CXCL10 treatment group compared to the control group (**Supplementary Figure 1C**). To confirm the essential role of CXCR3 in ROS accumulation, we used small interfering RNA (siRNA) to silence CXCR3 expression in the NK92 cell line, followed by CXCL10 stimulation. The results demonstrated significantly reduced ROS production in the si-CXCR3 group compared to the control (**Supplementary Figures 1D, E**). Following ROS suppression with NAC, there was a significant reduction in autophagy induced by ROS accumulation (**Figure 4B**).

Furthermore, we investigated the association between ROS levels and the severity of radiation pneumonitis using an animal model. As anticipated, the inhibition of ROS by NAC substantially ameliorated inflammatory lesions in lung tissues (**Figure 4C** and **Supplementary Figure 2A**).

To further investigate the direct regulatory impact of excessive autophagy on NK cell function, we performed the following experiments. First, we utilized a CCK-8 assay to evaluate the effect of autophagy on NK cell vitality. The results indicated that inhibiting radiation-induced excessive autophagy in NK cells with 3-MA led to a significant increase in their proliferation rate compared to the control group (**Figure 4D**). Subsequently, we evaluated alterations in NK cell function following the inhibition of radiation-induced excessive autophagy using flow cytometry. As anticipated, there was a significant recovery in NK cell function after autophagy inhibition (**Figure 4E**). To better illustrate the connection between NK cell function and autophagy, we simultaneously measured NK cell function and LC3 expression in the same samples and conducted a linear correlation analysis. The results demonstrated a significant negative correlation between the two variables (**Figure 4F**).

We further conducted *in vivo* experiments to confirm the influence of autophagy on NK cells. An experiment with two groups of mice (3-MA+RP vs RP) was designed. The results indicated that in the presence of 3-MA, there were no significant changes in the number of NK cells in mouse lungs (**Figure 4G**). This implies that autophagy may not directly control the quantity of NK cells, as the recruitment of NK cells induced by radiation is primarily governed by chemotactic factors. We then assessed the functionality of NK cells in the lungs of both groups of mice and observed that NK cell function in the RP+3-MA group significantly exceeded that in the RP-only group (**Figure 4H**). Targeting autophagy holds promise for restoring NK cell function impaired in radiation pneumonitis.

### Excessive autophagy leads to the internalization of NKG2D

**Figure 5:**
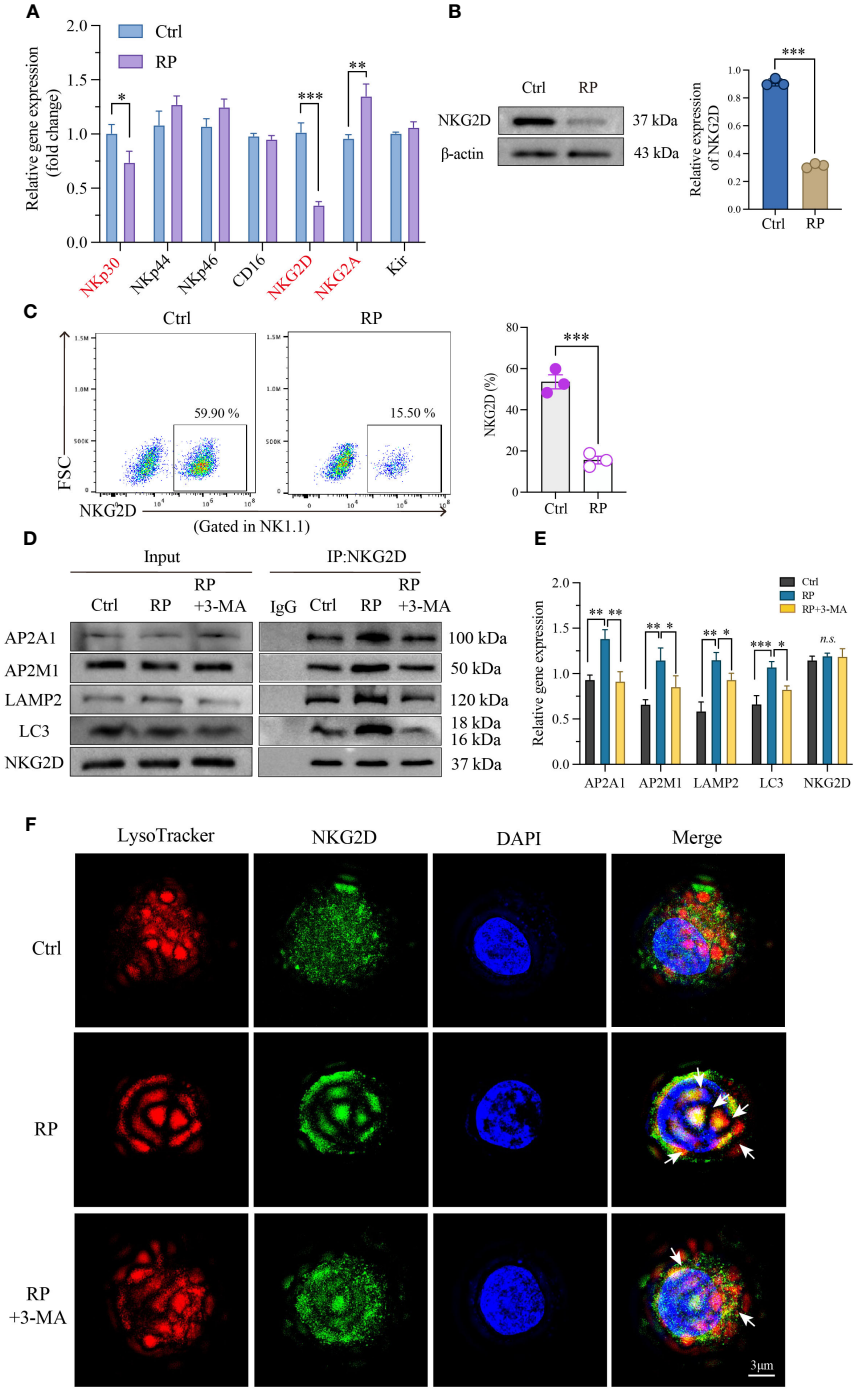
Excessive autophagy leads to the internalization of NKG2D. **(A)** Using RT-PCR technology to examine RNA expression of the main functional receptors on the NK cell surface. And the reduced NKG2D expression on the surface of NK cells was further confirmed by western blotting **(B)** and flow cytometry **(C)**. **(D, E)** Co-IP demonstrated a significant increase in the interaction between NKG2D and LAMP2 after radiation therapy, and inhibiting autophagy likewise attenuated their binding. **(F)** A reconstructed three-dimensional structured illumination microscopy (3D-SIM) image showing an NK cell. The cell surface is labeled with an antibody against NKG2D (green), and lysosomes are labeled with a specific lysosomal marker (red). The merged image demonstrates the co-localization of NKG2D and lysosomes (arrow) within the NK cell. Scale bar = 3 μm. A-E: n = 3. Each point represents an individual experiment. *, P < 0.050; **, P < 0.010; ***, P < 0.001. *P = 0.0236 NKp30, ***P = 0.0002 NKG2D, **P = 0.0044 NKG2A **(A)**, ***P < 0.0001 **(B)**, ***P = 0.0006 **(C)**, **P = 0.0021, **P = 0.0016, AP2A1; **P = 0.0039, *P = 0.0390, AP2M1; **P = 0.0060, *P = 0.0472, LAMP2; ***P = 0.0009, *P = 0.0116, LC3 **(E)**.

In our prior research, we confirmed the decline in NK cell function in radiation pneumonitis, although the precise mechanisms remain unknown. We employed RT-PCR to assess the RNA expression of key functional receptors on the NK cell surface, revealing a significant reduction in NKG2D receptor expression after radiotherapy compared to the control group (**Figure 5A**). The decreased NKG2D expression on NK cell surfaces after radiation was corroborated by western blot and flow cytometry, confirming the PCR findings (**Figures 5B, C**).

Next, we investigated the degradation mechanism of NKG2D. Immunoprecipitation studies showed enhanced interaction between NKG2D and the clathrin AP2 subunit after radiation therapy, which was blocked by 3-MA. AP2 is a crucial component in clathrin-mediated endocytosis, capable of binding to clathrin and membrane cargo proteins, thus playing a pivotal role in endocytosis. Conversely, LC3, a distinctive molecule in autophagosome formation, serves as a specific marker for autophagy. Moreover, LC3 lipidation can induce direct interaction with various autophagic components through cargo receptors. To ascertain the direct connection between NKG2D and autophagy, we further investigated the interaction between NKG2D and LC3, demonstrating an enhanced interaction between NKG2D and LC3 after radiation therapy. Furthermore, we noted a notable rise in the interaction between NKG2D and LAMP2 (a lysosomal marker protein) after radiation therapy, and the inhibition of autophagy similarly reduced their binding (**Figures 5D, E**).

To further explore the extent of autophagy in NK cells under different treatments, we utilized 3D-SIM (Structured Illumination Microscopy) technology to confirm autophagy at the organelle level. Our results indicated that after irradiation, there was an elevated binding of NKG2D (green) with lysosomes (red) compared to the control group, suggesting an increased co-localization. This effect was suppressed when irradiation was combined with the autophagy inhibitor 3-MA, providing a more direct and microscopic representation of the extent of autophagy within lysosomes (**Figure 5F** and **Supplementary Figure 2B**). These findings collectively indicate that radiation-induced excessive autophagy results in the degradation of the membrane protein NKG2D through a clathrin-lysosome-dependent pathway.

### Autophagy-NK axis regulates radiation pneumonitis and tumor progression

**Figure 6:**
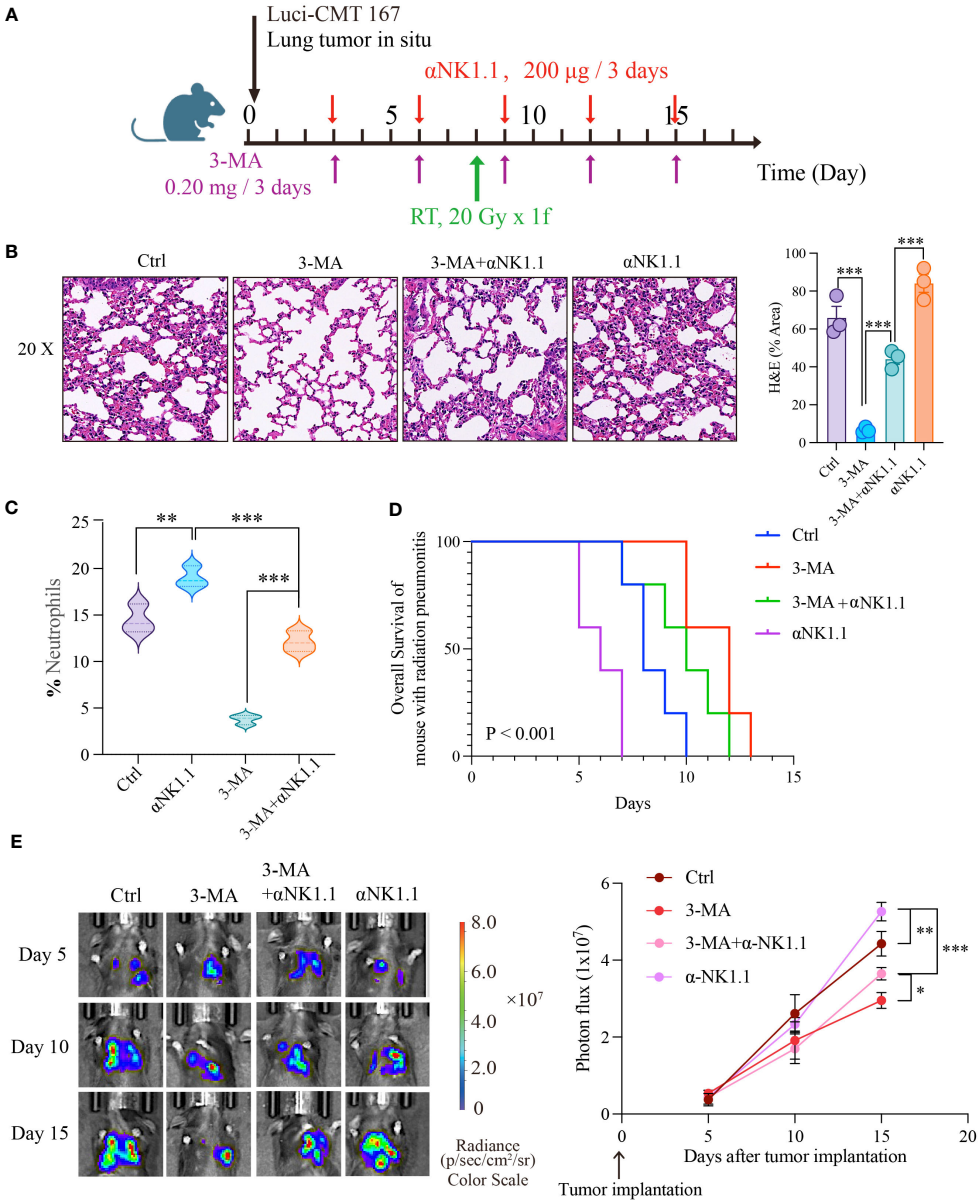
Autophagy-NK axis regulates radiation pneumonitis and tumor progression. **(A)** Animal model diagram. Evaluate the severity of radiation pneumonitis from three perspectives: HE of pulmonary tissue **(B)**, and the number of neutrophils in bronchoalveolar lavage fluid **(C)**. Then we examined the effect of autophagy and NK cells on tumor progression during radiotherapy using live animal imaging and survival time detection **(D, E)**. **(B, C)**: n = 3; **(D, E)**: n = 5. Each point represents an individual experiment. *, P < 0.050; **, P < 0.010; ***, P < 0.001. *** P < 0.0001 Ctrl vs 3-MA, ***P = 0.0008 3-MA vs 3-MA+αNK1.1, ***P = 0.0005 3-MA+αNK1.1 vs αNK1.1 **(B)**, **P = 0.0053 Ctrl vs αNK1.1, ***P = 0.0003 αNK1.1 vs 3-MA+αNK1.1, ***P < 0.0001 3-MA vs 3-MA+αNK1.1 **(C)**, **P = 0.0065 Ctrl vs αNK1.1, ***P < 0.0001 αNK1.1 vs 3-MA+αNK1.1, *P = 0.0242, 3-MA+αNK1.1 vs 3-MA **(E)**.

As widely recognized, NK cells play a crucial role not only in inflammation but also in anti-cancer therapy. In clinical practice, patients undergoing thoracic radiotherapy often have concurrent tumors, and lung cancer is particularly closely linked to radiation pneumonitis. We combined the radiation pneumonitis model with a mouse model of *in situ* lung cancer to investigate the combined impact of targeting autophagy and NK cell function on inflammation and cancer (**Figure 6A**) . Firstly, we assessed the severity of radiation pneumonitis from two angles: HE staining and the number of neutrophils in bronchoalveolar lavage fluid. Results showed that 3-MA significantly inhibited the progression of pneumonitis, but this inhibitory effect was noticeably reduced when NK cells were depleted. Furthermore, pneumonitis significantly worsened when NK cells were independently depleted (**Figures 6B, C**). Next, we employed live animal imaging and survival time detection to assess how autophagy and NK cells influence tumor progression during radiotherapy. Results demonstrated that the progression of tumors mirrored the severity of inflammation (**Figures 6D, E**). In addition, we also assessed the cytotoxicity<city/> of NK cells against tumor cells in an *in vitro* experiment under the backdrop of radiation pneumonitis. The results showed that compared to the control group, NK cells from the lungs of radiation pneumonitis mice exhibited significantly reduced cytotoxicity<city/> against the mouse lung cancer cell line CMT167 (**Supplementary Figures 2C, D**). This suggests that targeting autophagy and NK cells has a significant effect in slowing the progression of radiation pneumonitis, as well as in inhibiting tumor growth.

## Discussion

As a common early complication of chest radiation therapy, Radiation Pneumonitis (RP) can lead to treatment interruption, and even respiratory failure, significantly impacting patient prognosis and survival (2). Unfortunately, there is currently no effective medication for treating or preventing RP, and the specific pathogenesis of RP remains unclear, necessitating further investigation (20). NK cells, as integral components of the immune system, have a complex impact on inflammation. On one hand, NK cells, when in balanced quantities and functioning normally, can alleviate excessive inflammatory responses within the body; on the other hand, the excessive accumulation of NK cells can secrete a substantial amount of pro-inflammatory cytokines, intensifying the inflammatory response (21). NK cells also influence other immune cells in complex ways. For instance, NK cells regulate and enhance T cell immune responses by producing IFN-γ. Additionally, NK cells impact the maturation of macrophages by secreting IL-10 (5, 22).

**Figure 7:**
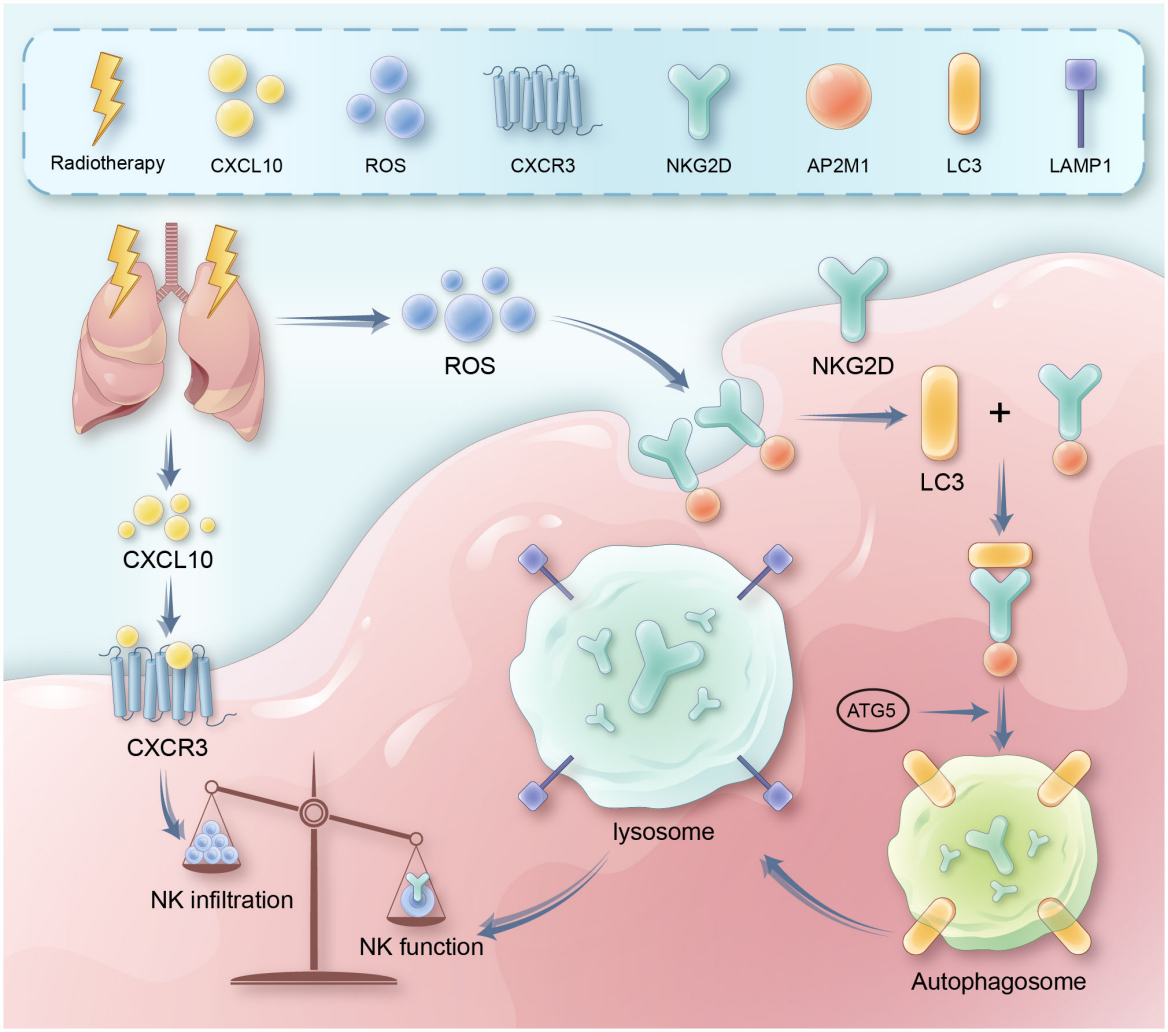
It presents a schematic diagram outlining the proposed mechanism. Key reactions, interactions, and resultant effects are represented with arrows. Radiation therapy induces DNA damage and cell death in lung tissue, leading to release of inflammatory cytokines like CXCL10. CXCL10 binds to and activates the CXCR3 receptor on natural killer (NK) cells, triggering signaling cascades involving RhoA and MAPK. This leads to recruitment and infiltration of more NK cells from circulation into the damaged lung tissue. Meanwhile, radiation also causes accumulation of reactive oxygen species (ROS) in lung tissue and NK cells. Excessive ROS induces overactivation of autophagy pathways in NK cells. This involves increased expression of autophagy proteins like LC3. Through adaptor proteins like AP2, LC3 binds to and internalizes the NKG2D activating receptor on NK cells, routing it for degradation in lysosomes. Loss of surface NKG2D impairs NK cell cytotoxicity<city/> and cytokine production, preventing efficient clearance of damaged cells and resolution of inflammation. Therefore, the CXCL10-CXCR3 axis and ROS-induced autophagy combine to both increase total NK cells yet suppress their functions in radiation pneumonitis.

Importantly, the role of NK cells in radiation pneumonitis remains unclear. In this context, our *in vivo* experiments revealed significant alterations in the quantity and function of NK cells within RP. These alterations are characterized by an increase in NK cell numbers and a loss of function. To delve into the role and specific mechanism of NK cells in radiation pneumonitis, we employed techniques such as Elisa and Western blot. We discovered that radiation-induced lung damage releases a substantial amount of CXCL10, which rapidly activates the primary chemotactic receptor, CXCR3, on the surface of NK cells, leading to a significant infiltration of NK cells into lung tissue. In contrast to the increase in quantity, radiation therapy results in an excessive accumulation of Reactive Oxygen Species (ROS) within the cells, causing overactive autophagy in NK cells. Mechanistically, we confirmed through techniques like co-immunoprecipitation (CO-IP) that autophagy mediates the endocytosis of the membrane-bound NKG2D via the adaptor protein 2 (AP2), resulting in a decrease in NKG2D expression in NK cells and, consequently, NK cell dysfunction. Our data also indicated a significant correlation between the expression of the active receptor NKG2D on the surface of NK cells and the level of autophagy. In summary, we observed alterations in the quantity and function of NK cells in RP and, regarding the mechanism, we discerned that the activation of the CXCL10/CXCR3 axis recruits a substantial number of NK cells and, to a certain extent, promotes the accumulation of intracellular ROS. A noteworthy point is that the ROS generated by the activation of the CXCL10/CXCR3 axis is limited, but it plays a significant role in the enrichment of NK cells. It is the accumulation of ROS due to cellular radiative damage that serves as the primary factor for excessive autophagy (**Figure 7**). Therefore, interventions targeting CXCR3 still require more in-depth research to support their clinical applications, while measures intervening in autophagy and NK cells are effective preventive strategies for RP, offering critical theoretical support for the early clinical intervention and treatment of RP.

The infiltration and functional abnormalities of Natural Killer (NK) cells represent typical characteristics of inflammatory diseases (23, 24). However, research on NK cells lags behind that of other immune cells, and reports on NK cells in radiation pneumonitis are particularly scarce. In our study, we observed impaired NK cell function due to radiation therapy. The activation of the CXCL10/CXCR3 axis exacerbated radiation pneumonitis by inducing excessive autophagy, thereby hindering NK cells from clearing damaged tissue cells and hyper-reactive inflammatory cells. One distinctive feature of NK cells lies in their rapid activation and direct functional exertion via receptor-ligand binding on their membrane surface. The balance between activating and inhibitory receptors ultimately dictates NK cell functional phenotypes. In this context, we primarily focused on NKG2D (an activating receptor) without delving into alterations in other receptors and their interplay with NKG2D. Furthermore, numerous studies have documented NKG2D downregulation following repeated ligand binding (25–27), leading to what is termed NKG2D exhaustion. Investigating how this phenomenon impacts NK cell function in radiation pneumonitis merits our thorough examination.

Numerous reports have shown that tissue damage can trigger an increase in the release of inflammatory factors and chemokines, recruiting immune cells to infiltrate the site of inflammation (28, 29). In our study, we observed that CXCL10/CXCR3 accelerated NK cell infiltration into lung tissue, leading to intracellular ROS accumulation. While various studies have confirmed the impact of different chemokines on ROS release, research specifically focused on CXCR3’s role in promoting ROS release remains limited, necessitating further investigation into the underlying mechanism. Research conducted by Victorelli S and colleagues demonstrated that senescent melanocytes’ SASP induces telomere dysfunction in a paracrine manner, thereby limiting the proliferation of neighboring cells through CXCR3-dependent mitochondrial ROS. Conversely, Li MX et al. reported that knocking down CXCR3 in murine paw cells significantly inhibited high glucose-induced decreases in cell viability, cell cycle arrest, and intracellular ROS production. These related studies underscore the evident connection between CXCR3 and ROS, making the investigation of their interplay in radiation pneumonitis a key focus of our ongoing research.

As widely recognized, ROS is a well-established trigger for autophagy (30), as confirmed in our research on radiation pneumonitis (RP). Given that radiotherapy modulates NK cell function by activating autophagy, we investigated the deeper relationship and specific interaction between autophagy and NKG2D. Through co-immunoprecipitation, we verified that autophagy induces NKG2D internalization and degradation. Additionally, we observed that radiation enhances NKG2D binding with clathrin, adaptor protein 2 (AP2A1 and AP2M1 subunits), LC3, and lysosomes. This implies that clathrin-mediated endocytosis plays a pivotal role in autophagy-induced NKG2D degradation. Other studies also suggest that adaptor proteins can link autophagic lysosomes to membrane-surface Claudin-2, promoting its internalization and degradation, thereby reinforcing intestinal barrier tight junctions, aligning with our findings (31). Autophagy in Alzheimer’s disease has been shown to target membrane-bound amyloid precursor protein (APP, the precursor of β-amyloid) for degradation (32), providing substantial support for autophagy-mediated degradation of membrane proteins via adaptor proteins. In conclusion, autophagy plays a crucial role in our research, and inhibiting autophagy is likely to be beneficial for radiation pneumonitis. 3-MA, as a common autophagy inhibitor, has demonstrated efficacy in reversing radiation-induced lung injury in our studies. However, in tumor cells, the use of 3-MA often inhibits tumor proliferation and increases their drug sensitivity (33, 34). Of course, there are many kinds of drugs that inhibit autophagy; phospho-chloroquine is also a commonly used autophagy inhibitor. Whether it can play a role in radiation pneumonitis remains to be further studied by us.

In summary, our research provides new insights into how radiation therapy regulates NK cell function through autophagy-mediated degradation of NKG2D. It’s worth noting that in clinical treatments, the dosage and duration of radiation therapy vary widely, and different doses and durations produce different effects. In terms of mechanisms, apart from autophagy, the inflammatory environment in radiation pneumonitis exerts complex effects on the quantity and function of NK cells. Other mechanisms still require further in-depth exploration. Future studies will further elucidate the fine-tuning of this mechanism, including CXCL10/CXCR3-mediated ROS release, its precise role in NKG2D endocytosis and autophagic degradation, as well as the interactive role of autophagy and endocytic pathways in regulating NK cell function and repairing lung injury.

## Data availability statement

The data presented in the study are deposited in the NCBI repository, accession number PRJNA1035231.

## Ethics statement

Ethical approval was not required for the studies on humans in accordance with the local legislation and institutional requirements because only commercially available established cell lines were used. The animal study was approved by the Institutional Animal Care and Use Committee of Qilu Hospital affiliated to Shandong University. The study was conducted in accordance with the local legislation and institutional requirements.

## Author contributions

Conception and design: RW, BC, and YT. Acquisition of data: XM, XZ, and DJ. Analysis and interpretation of data: HM and ZL. Writing and review of the manuscript: RW, BC, XM, XZ, and DJ. Revision of the manuscript and study supervision: BC and YT. All authors contributed to the article and approved the submitted version.
