# Supplementary material for: Autophagy-mediated NKG2D internalization impairs NK cell function and exacerbates radiation pneumonitis
Source: Front Immunol. 2023 Nov 24;14:1250920. doi: 10.3389/fimmu.2023.1250920 (PMC10704197; doi:10.3389/fimmu.2023.1250920)
Supplement: Supplementary file 1 [file Image_1.pdf]

Supplementary Figure 1

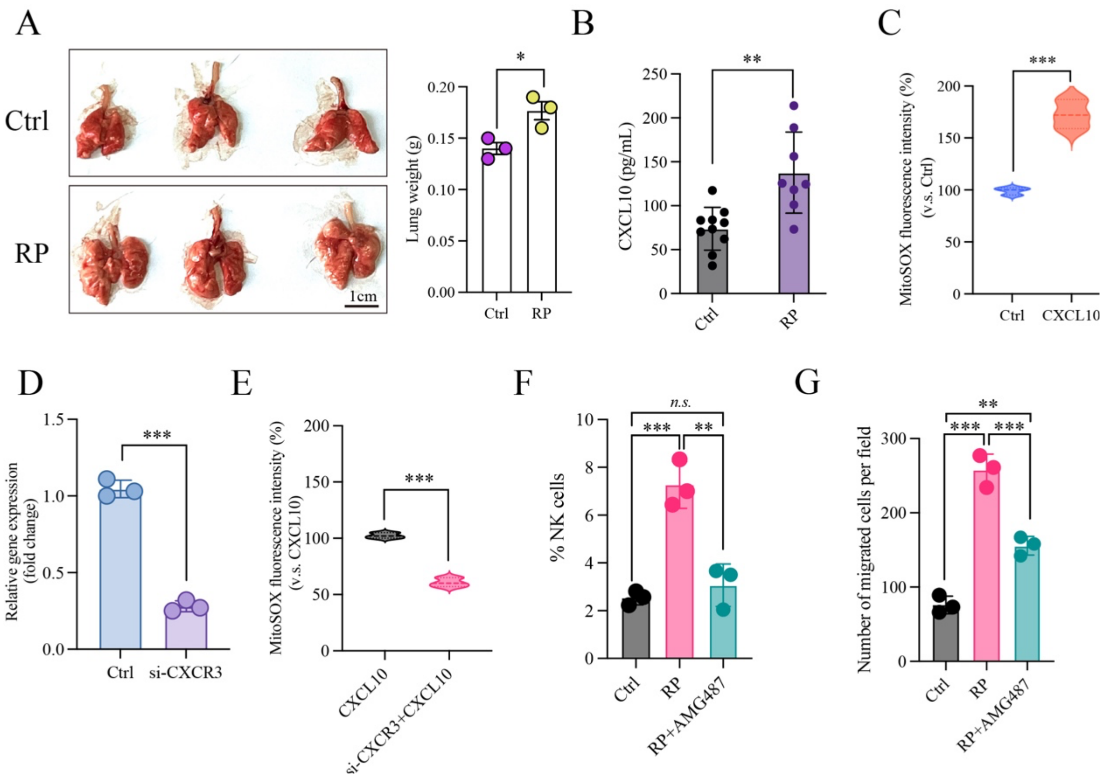

Supplementary Figure 1. (A) The image depicts gross anatomical views of lung tissue from control group and RP group mice. The histogram shows changes in the weight of lung tissue. **(B)** CXCL10 secretion was detected by ELISA in both groups. **(C, E)** Using mitochondrial ROS-specific fluorescent dye (MitoSOX red) to observe the distribution of mitochondrial ROS. **(D)** Knockdown of CXCR3 expression on the surface of NK cells. **(F)** Immunofluorescence statistical graph of NK cells after different treatments. **(G)** Statistical graph from the Transwell assay conducted on NK cells. A-F: n = 3. Each point represents an individual experiment. *, P < 0.050; **, P < 0.010; ***, P < 0.001.

Supplementary Figure 2

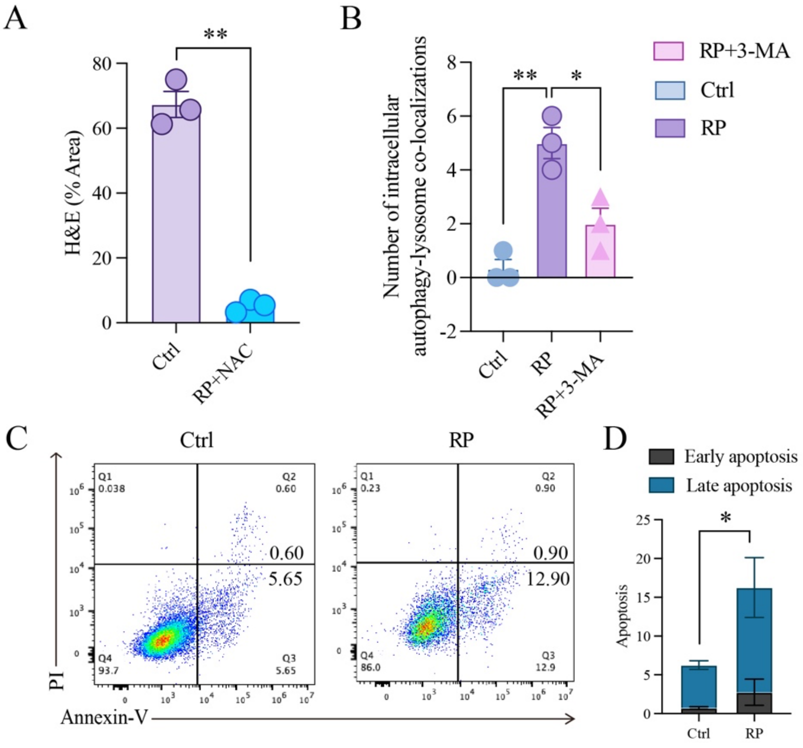

Supplementary Figure 2. (A) Statistical graph of H&E-stained lung tissue sections. **(B)** Statistical graph from 3D-SIM assay conducted on NK cells. **(C-D)** Apoptosis detection of tumor cells after co-culture with NK cells. A-D: n = 3. Each point represents an individual experiment. *, P < 0.050; **, P < 0.010; ***, P < 0.001.
