# Supplementary material for: Autophagy-mediated NKG2D internalization impairs NK cell function and exacerbates radiation pneumonitis
Source: Front Immunol. 2023 Nov 24;14:1250920. doi: 10.3389/fimmu.2023.1250920 (PMC10704197; doi:10.3389/fimmu.2023.1250920)
Supplement: Supplementary file 2 [file Table_1.docx]

**Supplementary Table 1. Primer Sequence**

| RT-qPCR | Primer | Sequence (5’3’) |
| --- | --- | --- |
| NKp46 | Forward | **ATGCTGCCAACACTCACTG** |
|  | Reverse | **ATGATGGGTTTCGGGAGAGTC** |
| CD16 | Forward | **TGGCAGCTACTACTACCAACA** |
|  | Reverse | **TTGGGGTCTAGGTTCACCACA** |
| NKG2D | Forward | **GCACTAACTACCAGTCAACCTG** |
|  | Reverse | **CTCGAACAACGAACATTGGAGA** |
| NKG2A | Forward | **GCCCCTGCAAAGGTTTTCC** |
|  | Reverse | **TCTGTGGGTTCTAGTCATTGAGG** |
| Kir | Forward | **TGGGACAGAATGTGACTCTTACA** |
|  | Reverse | **GCTCACTATGTGCTGACAATACA** |
| CCL3 | Forward | **TGTACCATGACACTCTGCAAC** |
|  | Reverse | **CAACGATGAATTGGCGTGGAA** |
| CCL4 | Forward | **TTCCTGCTGTTTCTCTTACACCT** |
|  | Reverse | **CTGTCTGCCTCTTTTGGTCAG** |
| CCL5 | Forward | **TTTGCCTACCTCTCCCTCG** |
|  | Reverse | **CGACTGCAAGATTGGAGCACT** |
| CXCL1  CXCL10 | Forward | **ACTGCACCCAAACCGAAGTC** |
|  | Reverse  Forward | **TGGGGACACCTTTTAGCATCTT**  **CCAAGTGCTGCCGTCATTTTC** |
|  | Reverse | **TCCCTATGGCCCTCATTCTCA** |
| CXCL12 | Forward | **TGCATCAGTGACGGTAAACCA** |
|  | Reverse | **CACAGTTTGGAGTGTTGAGGAT** |
